# Management and outcomes of brain metastases from pancreatic adenocarcinoma: a pooled analysis and literature review

**DOI:** 10.3389/fonc.2023.1326676

**Published:** 2024-01-08

**Authors:** Etienne Gouton, Marine Gilabert, Simon Launay, Elika Loir, Marguerite Tyran, Philippe Rochigneux, Olivier Turrini, Jonathan Garnier, Emmanuel Mitry, Brice Chanez

**Affiliations:** ^1^ Medical Oncology Department, Institut Paoli-Calmettes, Marseille, France; ^2^ Department of Medical Oncology, Centre Hospitalier Universitaire Vaudois (CHUV), Lausanne, Switzerland; ^3^ Radiotherapy Department, Institut Paoli-Calmettes, Marseille, France; ^4^ Digestive Surgery Department, Institut Paoli-Calmettes, Marseille, France; ^5^ Aix-Marseille Université, Marseille, France

**Keywords:** pancreatic cancer, brain metastases, brain surgery, machine learning, data mining, big data, literature review

## Abstract

**Background:**

Brain metastases (BM) are rare in pancreatic ductal adenocarcinoma (PDAC) and little data exists concerning these patients and their outcomes.

**Aim:**

We aimed to analyze the management, practices, and outcomes of patients presenting BM from PDAC both in our institution and in all cases reported in the literature.

**Methods:**

We conducted a retrospective, monocentric analysis using a data mining tool (ConSoRe) to identify all patients diagnosed with PDAC and BM in our comprehensive cancer center (Paoli-Calmettes Institute), from July 1997 to June 2022 (cohort 1). Simultaneously, we reviewed and pooled the case reports and case series of patients with PDAC and BM in the literature (cohort 2). The clinical characteristics of patients in each cohort were described and survival analyses were performed using the Kaplan-Meier method.

**Results:**

In cohort 1, 19 patients (0.3%) with PDAC and BM were identified with a median age of 69 years (range: 39-81). Most patients had metastatic disease (74%), including 21% with BM, at diagnosis. Lung metastases were present in 58% of patients. 68% of patients had neurological symptoms and 68% were treated by focal treatment (surgery: 21%, radiotherapy: 42%, Gamma Knife radiosurgery: 5%). In cohort 2, among the 61 PDAC patients with BM described in the literature, 59% had metastatic disease, including 13% with BM at diagnosis. Lung metastases were present in 36% of patient and BM treatments included: surgery (36%), radiotherapy (36%), radiosurgery (3%), or no local treatment (25%). After the pancreatic cancer diagnosis, the median time to develop BM was 7.8 months (range: 0.0-73.9) in cohort 1 and 17.0 months (range: 0.0-64.0) in cohort 2. Median overall survival (OS) in patients of cohort 1 and cohort 2 was 2.9 months (95% CI [1.7,4.0]) and 12.5 months (95% CI [7.5,17.5]), respectively.

**Conclusion:**

BM are very uncommon in PDAC and seem to occur more often in younger patients with lung metastases and more indolent disease. BM are associated with poor prognosis and neurosurgery offers the best outcomes and should be considered when feasible.

## 1 Introduction

With new efficient drugs lacking and a dismal prognosis, pancreatic ductal adenocarcinoma (PDAC) is estimated to become the second leading cause of cancer death by 2030 (1, 2). Currently, the majority of PDACs are diagnosed at a metastatic stage (mPDAC) with the most common metastatic sites being the liver (76%), lung (20%), lymph node (9.4%), and bone (6.8%) (3). Brain metastases (BM) are the most common intracranial tumors (4, 5) and occur mainly in lung cancer (19.9%), melanoma (6.9%), kidney cancer (6.5%), and breast cancer (5.1%) (6). They are associated with poor prognosis and are fatal in 18% to 50% of cases (7, 8). Regarding gastrointestinal (GI) cancers, the incidence of BM depends mainly on the primary tumor (9). Barnholtz-Sloan et al. revealed that colorectal cancer was the most common GI cancer to cause BM, with an incidence of approximatively 1.8% (5). Specific biological features have been identified for these GI-derived BM, such as HER2 overexpression or the impairment of DNA repair systems, e.g., defects in homologous repair (HRD) or mismatch repair (MMRd) (10, 11).

Regarding patients with PDAC, the incidence of BM is even less, occurring in only 0.25% to 0.6% of cases (3, 9, 12), probably as a consequence of either the unsually reported neurological symptoms in that disease or the short survival time of PDAC patients. Furthermore, brain imaging is not routinely recommended for the diagnosis of PDAC, thus some patients may have undetected asymptomatic BM. This hypothesis is supported by post mortem examinations of patients with PDAC, which reported a BM rate of 7.9% (13).

Therefore, little is known about the characteristics and therapeutic management of these patients as only case reports or small series have been reported thus far. In our retrospective study, we aimed to describe the management of all patients with BM from metastatic PDAC that were treated at the Paoli-Calmettes Institute, in Marseille, France (cohort 1). We then performed a comprehensive literature review and described the characteristics and clinical outcomes of a population pooled from these reported cases (cohort 2).

## 2 Materials and methods

### 2.1 Study population: cohort 1

A single-center retrospective analysis was conducted at the Paoli-Calmettes Institute (IPC, Marseille, France) to identify all patients diagnosed with both PDAC and BM during their cancer history. Said patients were identified via the clinical data mining interface ConSoRe (Continum Soin Recherche)—a tool for semantic search associated with a clinical data warehouse (French ConSoRe project)—using the following keywords: “pancreas” as primary tumor and “brain” as metastases location (14). Between July 1997 and June 2022, all patients ≥ 18 years presenting brain metastasis during the evolution of PDAC were recorded. Patients with pancreatic neuroendocrine tumors and patients with leptomeningeal disease without parenchymal tumors were excluded. All medical files of patients selected by ConSoRe were manually reviewed.

For each patient, clinical, biological, and radiological treatment data and outcomes were collected retrospectively after obtaining informed consent. This study was approved by our institution’s Institutional Review Board as n° *PDAC-MC-IPC 2022-035* and the study protocol conforms to the ethical guidelines of the 1975 Declaration of Helsinki (6th revision, 2008) as reflected in *a priori* approval by the institution’s human research committee.

### 2.2 Study population: cohort 2

A medical literature review was performed via the Pubmed and Google Scholar databases until June 2022, using the following keywords: “pancreatic adenocarcinoma”, “pancreatic neoplasms”, “pancreatic cancer”, “PDAC”, “brain metastasis”, “brain metastases”, “brain tumor”, and “CNS tumor”. Case reports, case series, and abstracts in English were considered. These articles’ references were also reviewed in order to locate additional cases. Reports of lesions discovered post-mortem were excluded. Clinical, biological, and radiological patient characteristics, as well as all available survival data, were also collected.

### 2.3 Objectives and statistical analysis

The primary objective of our study was to describe the characteristics and outcomes of patients with PDAC and BM from cohort 1 and compare these to those of the patients in cohort 2. Descriptive statistics were used to analyze patient characteristics. Overall survival (OS) was defined as the time from diagnosis of PDAC (pOS) or BM (bmOS) to death from any cause, censored at the date of last follow-up. Survival analyses were estimated via the Kaplan-Meier method, and differences between treatment groups in each cohort were tested via the log-rank test. We did not perform any statistical comparison between the two cohorts due to missing data in cohort 2 and great heterogeneity between both cohorts in terms of patient characteristics and treatments. Thus, we reported data from cohort 1 and cohort 2 in a side-by-side method. Statistical analyses were reviewed by our institution’s Biomedical Statistics Department. Statistical analyses were performed on IBM SPSS Statistics software, version 26.0.0.0 (IBM SPSS Inc., Chicago, IL, USA). A *p*-value<0.05 was considered as statistically significant.

## 3 Results

### 3.1 Clinical characteristics and treatments of patients with BM in cohort 1

Among the 6,113 patients diagnosed with PDAC at our institution between July 1997 and June 2022, we identified 19 patients with brain metastases (0.3%). Their characteristics are listed in **Table 1**. The median age at BM diagnosis was 69 years (range: 39-81) and primary tumors were located in the head (63%), tail (26%), or body (11%) of the pancreas. Two patients were tested for germline *BRCA* status, and one had a germline *BRCA1* mutation. Cancer genome sequencing was performed in only two patients: one patient with an *MSH6* mutation and one patient with a *KRAS G12V* mutation. Most patients were initially diagnosed with metastatic PDAC (74%). Prior to BM diagnosis, patients had received a median of one line of systemic chemotherapy (range: 0-3) and 37% had received at least two lines of chemotherapy.

**Table 1:** Patient characteristics.

Characteristics	Cohort 1		Cohort 2
	*n = 19*		*n = 61*
Sex			
Male	10 (53%)		36 (59%)
Female	9 (47%)		19 (31%)
Missing	0		6 (10%)
Age at BM diagnosis			
Median, years (range)	69 (39 – 81)		58 (35 – 78)
Initial PDAC stage			
Metastatic	14 (74%)		36 (59%)
*Including BM at diagnosis*	4 (21%)		8 (13%)
Concurrent metastatic sites			
Lung	11 (58%)		22 (36%)
Liver	11 (58%)		33 (54%)
Lymph nodes	5 (26%)		12 (20%)
Other	6 (32%)		11 (18%)
Number of systemic lines before BM diagnosis			
Median (range)	1 (0 – 3)		2 (0 – 3)
Missing	0		1
Number of systemic lines after BM diagnosis			
Median (range)	0 (0 – 2)		0 (0 – 3)
Missing	1		32

BM, brain metastases; PDAC, pancreatic ductal adenocarcinoma.

Percentages may not total 100 due to rounding.

Symptoms resulted from BM in 13 patients (68%) (**Table 2**). The most common neurological symptoms were confusion (26%) and symptoms of intracranial hypertension including headaches (21%) and motor disorders (16%). Notably, 37% had a single brain metastasis and 21% had more than 4, located exclusively in the supratentorial area in 74% (**Table 2**). Pathological analysis of BM was available in four patients (21%), with confirmation of the primary pancreatic origin. At BM diagnosis, extracranial disease was stable in 32% and progressive in 21% of patients. One patient had a persistent complete extracranial response.

**Table 2:** Characteristics and local treatments of brain metastases.

Characteristics	Cohort 1	Cohort 2
	*n = 19*	*n = 61*
BM indications		
Neurological symptoms	13 (68%)	34 (56%)
Systematic brain imaging	3 (16%)	2 (3%)
Missing	3 (16%)	25 (41%)
Associated leptomeningeal carcinomatosis		
Yes	1 (5%)	6 (10%)
Neurological symptoms		
Confusion	5 (26%)	3 (5%)
Headache/ICH	4 (21%)	25 (41%)
Motor alterations	3 (16%)	17 (28%)
Cranial nerve dysfunction	2 (11%)	1 (2%)
Sensitivity alterations	0	4 (7%)
Stroke	1 (5%)	0
Dysarthria	0	4 (7%)
Dysphasia	2 (11%)	5 (8%)
Seizure	1 (5%)	6 (10%)
Visual impairments	0	8 (13%)
Ataxia	0	7 (11%)
None	3 (16%)	3 (5%)
Missing	3 (16%)	0
Number of BM		
1	7 (37%)	22 (36%)
2 – 3	4 (21%)	6 (10%)
≥ 4	4 (21%)	8 (13%)
Missing	4 (21%)	25 (41%)
BM location		
Supratentorial	14 (74%)	37 (61%)
Infratentorial	0	6 (10%)
Both	4 (21%)	16 (26%)
Missing	1 (5%)	2 (3%)
Extracranial status at BM diagnosis		
Complete response	1 (5%)	7 (11%)
Partial response	0	3 (5%)
Stable disease	6 (32%)	0
Progressive disease	4 (21%)	10 (16%)
Initial diagnosis	5 (26%)	5 (8%)
Missing	3 (16%)	36 (59%)
Local treatment of BM		
Surgery	4 (21%)	22 (36%)
Radiotherapy	8 (42%)	22 (36%)
*Including WBRT*	6 (32%)	16 (26%)
Radiosurgery	1 (5%)	2 (3%)
Best supportive care	5 (26%)	15 (25%)
Missing	1 (5%)	2 (3%)

BM, brain metastases; ICH, intracranial hypertension; WBRT, whole-brain radiotherapy. Percentages may not total 100 due to rounding.

Four patients (21%) underwent surgery for BM (**Table 2**), among which three received postoperative radiotherapy. Exclusive radiotherapy was performed in 42% of patients with whole-brain radiotherapy (WBRT) in six patients (32%) and standard or stereotactic radiotherapy in two patients (11%). One patient (5%) received stereotactic radiosurgery and 1 patient had missing data (5%). Five patients (26%) did not receive any local treatment for BM.

### 3.2 Clinical characteristics and treatments of patients with BM in cohort 2

Based on our literature review, 24 articles were found, including 61 patients (**Supplementary Table S1**, **Supplementary Materials**) (15–38). The pooled patient characteristics are described in **Table 1**. Patients of cohort 2 were younger at BM diagnosis than those in cohort 1 (58 years *vs* 69 years, respectively). Among six patients with known germline *BRCA* status in cohort 2, three patients had a germline *BRCA1* (*n*=1) or *BRCA2* (*n*=2) mutation, all reported by Jordan et al. (29). One additional patient displayed a somatic *BRCA2* mutation in both the primary pancreatic tumor and BM (38). At initial diagnosis, 25 patients (41%) had localized PDAC. Patients had received a median of two prior lines of chemotherapy (range: 0-3). The most common metastatic sites were the liver (54%), lung (36%), lymph nodes (20%), and peritoneum (11%).

Regarding BM, the most frequent symptoms were those of intracranial hypertension including headaches (41%), motor alteration (28%), and visual impairment (13%). Patients were less often confused in cohort 2 than in cohort 1 (5% *vs* 26%, respectively). Cystic lesions constituted BM in 16% of patients and BM were localized mainly in the supratentorial area (61%), infratentorial area (10%), or both (26%). Of note, 16% of patients had discordant evolution between intra and extracranial lesions; 11% and 5% had complete and partial extracranial responses at BM diagnosis, respectively.

In cohort 2, BM treatments comprised surgery (36%), radiotherapy (36%, of which 26% and 8% received WBRT and standard/stereotactic radiotherapy, respectively), radiosurgery (3%), and no local treatment (25%). Among the 22 surgery patients, 13 (59%) received postoperative irradiation of the tumor bed.

### 3.3 Survival analysis of patients with PDAC and brain metastases

**Figure 1:**
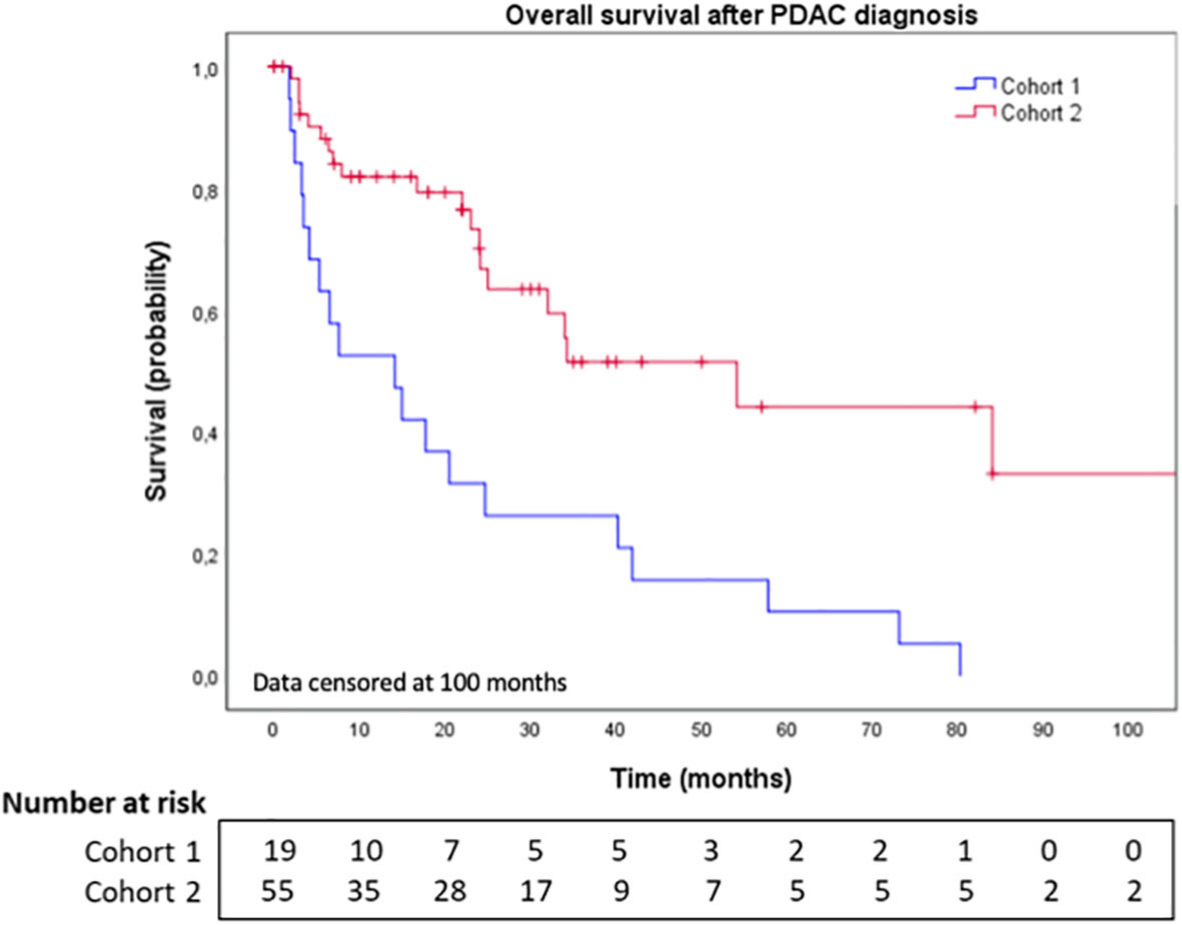
Overall survival after brain metastases diagnosis in cohort 1 and cohort 2. BM: Brain metastases. Data censored at 100 months.

**Figure 2:**
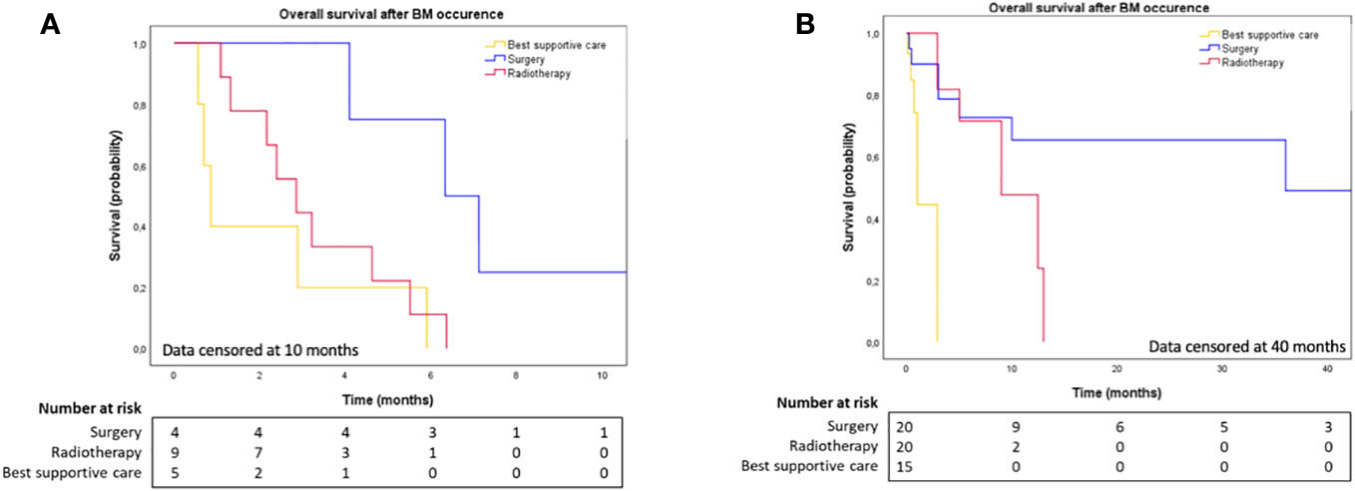
**(A)** Overall survival after brain metastases diagnosis in cohort 1, according to BM local treatment. Data censored at 10 months. **(B)** Overall survival after brain metastases diagnosis in cohort 2, according to BM local treatment. Data censored at 40 months.

The median time to BM diagnosis after PDAC diagnosis in cohorts 1 and 2 was 7.8 months (range: 0.0-73.9) and 17.0 months (range: 0.0-64.0), respectively (**Table 3**). At the end date of follow-up (July 4^th^, 2022), all patients from cohort 1 had died. **Figure 1** illustrates OS for both cohorts. After BM surgery, patients in cohorts 1 and 2 experienced a bmOS of 6.3 months (95% CI [3.4, 9.3]) and 36.0 months (95% CI [NR, NR]) respectively (**Figures 2A, B**). After surgery, the median time to recurrence was 4.1 months (range: 3.9-4.3) and 8 months (range: 4.0-12.0) in cohorts 1 and 2, respectively (**Table 3**). Despite recurrences, five patients (from both cohorts 1 and 2) still benefited from surgery, as they displayed an overall median bmOS of 21.7 months (95% CI [0.0, 44.7]).

**Table 3:** Time to brain metastases and outcomes.

Outcomes	Cohort 1	Cohort 2
	*n = 19*	*n = 61*
Time from PDAC diagnosis to BM diagnosis		
Median, months	7.8	17
Range	0.0 – 73.9	0.0 – 64.0
Overall survival after PDAC diagnosis		
Median, months	14.1	54.1
95% CI	2.1 – 26.2	18.5 – 89.7
Overall survival after BM diagnosis		
Median, months	2.9	12.5
95% CI	1.7 – 4.0	7.5 – 17.5
BM recurrence		
*n* (%)	2 (11%)	3 (5%)
Median, months	4.1	8
Range	3.9 – 4.3	4.0 – 12.0

BM, brain metastases; PDAC, pancreatic ductal adenocarcinoma.

### 3.4 Neurosurgery in patients from both cohort 1 (n=4) and cohort 2 (n=22)

In patients that underwent BM surgery, the median age at BM diagnosis was 61 years in both cohorts. The median time from PDAC diagnosis to BM diagnosis was 21.7 months (range: 0-36.0) and 17.0 months (range: 0-64) in cohorts 1 and 2, respectively. In cohort 1, neurosurgery was performed in a median time of 6.5 days after BM diagnosis and the median OS after surgery was 5.9 months (95% CI [3.7, 8.2]). Because of missing data, analysis of BM surgery was not conducted in cohort 2, only survival analyses from BM diagnosis.

Three patients (75%) in cohort 1 and 12 patients (55%) in cohort 2 had metastatic PDAC at initial diagnosis, including BM for four of them (one in cohort 1, three in cohort 2). In cohort 1, one patient had an extracranial complete response after chemotherapy and BM were the only metastatic recurrence. Other patients had lung (50%), liver (50%), lymph nodes (25%), and bone (25%) metastases. In cohort 2, 10 patients (45%) had no extracranial disease and the most common metastatic sites were the liver (23%), lymph nodes (9%), and lung (5%).

There were no major postoperative complications in either cohort; only one patient from cohort 1 had a transitory aphasia that resolved rapidly. Postoperative irradiation of the tumor bed was performed in 16 patients (62%): three patients (75%) in cohort 1 and 13 patients (59%) in cohort 2.

### 3.5 Case of a patient with germline BRCA1-mutated PDAC and BM with a long survival after surgery

A 57-year male patient in cohort 1 was diagnosed in October 2015 with a lesion in the head of the pancreas and synchronous multiple hepatic metastases. The patient had a family history of cancer (grandmother with pancreatic cancer, mother with breast cancer) and a germline *BRCA1* mutation was identified. The patient achieved a complete response after the first line of a FOLFIRINOX regimen followed by maintenance therapy with capecitabine.

**Figure 3:**
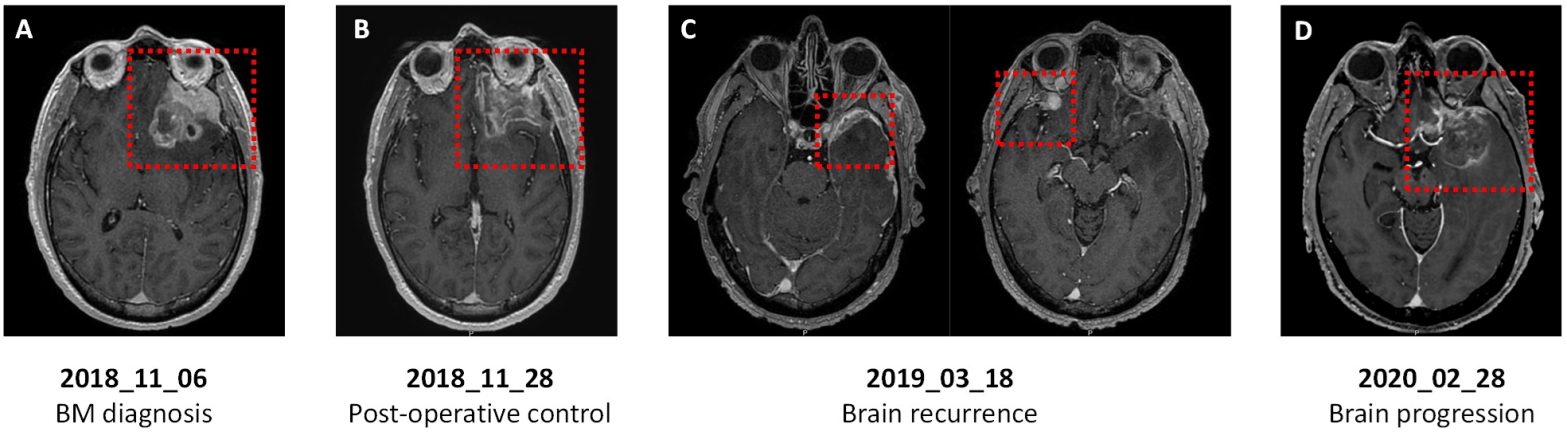
Axial T1-weighted brain magnetic resonance imaging (MRI) of a patient with PDAC and germline BRCA1 mutation. **(A)** At BM diagnosis: large left anterior frontal tumor in contact with the left optic nerve and perilesional edema. **(B)** After surgery: left fronto-orbital and dorsolateral ablation site without intraparenchymal enhancement. **(C)** At first brain recurrence: left frontal nodular meningeal tissue lesion (left) and new right inferior frontal enhanced nodular lesion with meningeal implantation (right). **(D)** At second brain recurrence: large left temporopolar and frontobasal lesion with significant perilesional edema.

In October 2018, the patient presented with headache without neurological impairment. Although a CT-scan indicated a persistent extracranial complete response, brain MRI revealed a > 5cm left anterior frontal tumor in contact with the left optic nerve, perilesional edema, and compression of the left lateral ventricle (**Figure 3A**). The patient underwent complete surgical resection of the BM in November 2018 (**Figure 3B**), reporting a CK7 positive, CK20 and TTF-1 negative adenocarcinoma consistent with the primary pancreatic origin. Postoperative stereotactic irradiation of the tumor bed was performed. Due to the absence of extra and intracranial residual disease, simple monitoring was proposed.

The patient relapsed in March 2019 with local meningeal recurrence at the surgical site and a left temporal lesion (**Figure 3C**). These lesions were treated with stereotactic irradiation, then systemic therapy with FOLFOX was resumed for a new extracranial adrenal lesion. A partial response was achieved until February 2020, when a new brain tumor of the left frontotemporal lesion was identified (**Figure 3D**). The patient underwent a second brain surgery in March 2020 and chemotherapy was pursued. After three additional cycles, hepatic and peritoneal progression was detected, and the patient died from PDAC-related complications in August 2020, 58 months after initial PDAC diagnosis and 22 months after first BM occurrence.

## 4 Discussion

Our study evaluated clinical characteristics and outcomes of patients with BM from PDAC using a single institution cohort of 19 patients and a pooled analysis of the literature review and aimed to provide a global overview of this rare and dreadful form of metastatic cancer.

Here, we confirm that BM are uncommon in pancreatic cancer, as previously reported` in the SEER PDAC database with BM occurring in only 0.6% of cases (90 out of 13,233) (3), or as reported by Jordan et al. with 0.4% of BM out of 5,824 mPDAC cases between 2000 and 2016 (29). In our study, 0.3% of patients with mPDAC disease were diagnosed with BM over a 25-year time period. However, the incidence of BM may be underestimated as suggested by post-mortem reports (13, 39), mainly due to the poor prognosis associated with extracranial disease; patients do not have “time” to develop neurological symptoms and therefore BM remain asymptomatic and unknown. Furthermore, due to the low incidence of BM in pancreatic cancer, routine brain imaging is only recommended by international guidelines if symptoms occur in the initial evaluation of pancreatic cancer (40, 41).

In our study, 58% and 36% of patients in cohorts 1 and cohort 2 had lung metastases, respectively. Such incidence is higher compared to the 20% rates published in epidemiological studies of mPDAC (3). Conversely, patients in our study had less frequent liver metastases (58% in cohort 1 and 54% in cohort 2) than usual in PDAC patients without BM (76%) (3). Interestingly, it has been demonstrated that lung and liver metastases in PDAC are associated with better and worse survival, respectively (3, 42). Hence, a more indolent disease in patients with lung metastases and no liver metastases could have more time to develop BM. Sasaki et al. also suggested that lung metastases could be a risk factor for the development of BM in patients with PDAC (31). Moreover, the patients in our study were younger than the usual median age of 71 years old at PDAC diagnosis (43), with a median age of 69 years and 58 years in cohorts 1 and 2, respectively. Data analysis of over 126,066 patients with pancreatic cancer from the SEER database revealed that younger patients (< 40 years) had significantly better OS than older patients aged 40-60 years (HR = 1.86; 95% CI [1.72, 2.01]; *p*<0.0001), 60-80 years (HR = 2.22; 95% CI [2.05, 2.40]; *p*<0.0001), and over 80 years (HR = 3.30; 95% CI [3.05, 3.57]; *p*<0.0001) (44). Altogether, these data highlight a patient profile of those more susceptible to developing BM, such as young patients with lung metastases and more likely indolent disease.

Additionally, a *BRCA* mutation (germline or somatic) was identified in 5 patients in the overall population of both cohorts, representing approximatively half of patients with an available *BRCA* status. This is a relatively high incidence considering that very few patients had genomic data available in our study. In mPDAC, a *BRCA* mutation is associated with an improved response to platinum-based chemotherapy and better survival (45–47). Interestingly, *BRCA* mutations have been associated with an increased risk of BM in both ovarian and breast cancer (48, 49). While further study is required in mPDAC, *BRCA* mutations may lead to increased BM development in PDAC. We also reported the case of a patient with a germline *BRCA1*-mutated PDAC who achieved a 3-year complete response to chemotherapy though BM occurred—and was surgically removed—and who also achieved an extracranial response that persisted for four years, indicating an indolent and chemotherapy-sensitive BRCA-mutated disease.

The management of BM is variable according to the European Association of Neuro-Oncology (EANO) guidelines (50). Surgery should be considered for a limited number of metastases (one to three), especially in cases of large (≥ 3 cm), symptomatic, or complicated (edema, necrosis, posterior fossa location with obstructive hydrocephalus) BM. Stereotactic radiosurgery should be offered to patients with either a limited number of BM (one to four) or a higher number (five to ten) but with a total tumor volume < 15 ml. The utilization of WBRT as an option for multiple BM is widespread yet currently disputed due to significant neurocognitive toxicities (51, 52). Nevertheless, best supportive cares remain a valid option for patients with poor ECOG performance status.

Our results suggest that in PDAC, BM surgery was both well-tolerated with no postoperative issues and associated with improved survival compared to radiotherapy or BSC (**Figure 2**). This finding is consistent with previous reports of several patients with BM from PDAC who experienced improved survival after neurosurgery. For example, Lemke et al. reported two cases of patients initially treated for localized PDAC who developed a single metachronous brain metastasis. Both had BM surgery, and no recurrence or other systemic metastases occurred during follow-up. The survival time of these two patients were 136 months and 74 month, and they were still alive when the case report was published (22). This emphasizes the importance of surgery in brain oligometastases. One of our patients from cohort 1, with a *BRCA1* mutation, underwent surgery for a single BM and achieved a prolonged survival of 21.7 months after the first BM resection. Overall, brain surgery appears feasible in selected patients and can provide improved survival.

While the central nervous system’s (CNS) involvement is uncommon in PDAC, other rare neurological locations have been reported. Choroidal metastases are also an exceptional event, as Shah et al. reported an incidence of 0.4% (53). Leptomeningeal metastases related to pancreatic cancer have also been reported, to our knowledge, in only 12 patients (54). One of these, reported by Rao et al., was also included in our review because of the association of multiple supratentorial and cerebellar hemispheric lesions (24). Although patients with isolated carcinomatous meningitis were not included in our study, one patient with leptomeningeal metastasis associated with BM was identified in our institutional database. In this patient, BM were diagnosed 39 months after the initial diagnosis of PDAC and the patient died only 2 weeks after the BM diagnosis.

Our study has several limitations. First, it is a retrospective, single-center study, with all the associated biases. Moreover, due to large amounts of missing data and great heterogeneity in reported cases, which would have caused biases, we did not perform any statistical comparison between the two cohorts. However, a large difference in survival appears to exist between the two cohorts (bmOS: 2.9 months in cohort 1 *vs* 12.5 months in cohort 2) that may be explained by i) the selective bias associated with case reports where usually the more favorable cases are published and ii) greater access in our center to brain imagery and neurosurgeons that could enlarge treatment indications. Moreover, in the largest study published investigating BM from PDAC, *Jordan et al.* (29) described 25 patients with a median bmOS of 1.9 months. Their study highlights the importance of analyses based on unselected patient cohorts like our cohort 1. Another limitation is the lack of molecular profiling available for most patients (cohort 1 and cohort 2) that limits the analysis of specific genomic profiles for BM from PDAC. However, *BRCA* mutations could be frequently associated to BM and brain CT-scan or MRI could be considered in that population when neurological symptoms or at disease progression. Certain data on genomic status in PDAC with BM describe the frequent alteration of *KRAS*, which is usual in PDAC (29), as well as a case of an *ALK* rearrangement–positive PDAC with BM that was sensitive to the *ALK* tyrosine-kinase inhibitor crizotinib after chemotherapy resistance and was also responsive to alectinib after BM diagnosis (36).

## 5 Conclusion

In conclusion, the occurrence of BM remains a very rare event in pancreatic cancer. A profile of patients susceptible to developing BM could be drawn from our results, mostly in indolent and slowly progressive disease (younger patients, lung metastases, BRCA mutations). However, additional studies are required to further explore molecular and/or genetic specificities. Improved survival in patients who underwent brain surgery was also clearly shown and thus, when feasible, should be proposed as the most personalized treatment.

## Data availability statement

The original contributions presented in the study are included in the article/**Supplementary Material**. Further inquiries can be directed to the corresponding author.

## Ethics statement

The studies involving humans were approved by IRB _ Paoli Calmettes Institute. The studies were conducted in accordance with the local legislation and institutional requirements. The participants provided their written informed consent to participate in this study. Written informed consent was obtained from the individual(s) for the publication of any potentially identifiable images or data included in this article.

## Author contributions

EG: Data curation, Formal Analysis, Investigation, Methodology, Resources, Software, Visualization, Writing – original draft, Writing – review & editing. MG: Conceptualization, Supervision, Validation, Writing – original draft, Writing – review & editing, Visualization. SL: Writing – review & editing, Data curation, Investigation. EL: Data curation, Investigation, Writing – review & editing. MT: Data curation, Writing – review & editing. PR: Data curation, Writing – review & editing, Formal Analysis, Methodology. OT: Writing – review & editing. JG: Data curation, Writing – review & editing. EM: Data curation, Formal Analysis, Writing – review & editing. BC: Conceptualization, Data curation, Formal Analysis, Investigation, Methodology, Project administration, Supervision, Validation, Writing – original draft, Writing – review & editing.
