# Supplementary material for: Management and outcomes of brain metastases from pancreatic adenocarcinoma: a pooled analysis and literature review
Source: Front Oncol. 2024 Jan 8;13:1326676. doi: 10.3389/fonc.2023.1326676 (PMC10800932; doi:10.3389/fonc.2023.1326676)
Supplement: Supplementary file 1 [file DataSheet_1.pdf]

SUPPLEMENTARY MATERIALS

Table S1: Case reports and series included in the literature review (cohort 2)

**Authors**	**Year**	**Number of patients** *(n)*	**Reference**
Kuratsu *et al.*	1990	2	(15)
Park *et al.*	2003	4	(16)
El Kamar *et al.*	2004	1	(17)
Caricato *et al.*	2006	1	(18)
Marepaily *et al.*	2009	1	(19)
Matsumura *et al.*	2009	1	(20)
Zaanan *et al.*	2009	1	(21)
Lemke *et al.*	2011	2	(22)
Chiang *et al.*	2012	1	(23)
Rao *et al.*	2013	1	(24)
Rajappa *et al.*	2013	1	(25)
Kumar *et al.*	2015	5	(26)
Matsumoto *et al.*	2015	1	(27)
Tardivo *et al.*	2018	1	(28)
Jordan *et al.*	2018	25	(29)
Lee *et al.*	2018	1	(30)
Sasaki *et al.*	2019	2	(31)
Matsuo *et al.*	2019	1	(32)
Luu *et al.*	2019	4	(33)
Dalal *et al.*	2019	1	(34)
Oka *et al.*	2021	1	(35)
Ou *et al.*	2021	1	(36)
Papadimitriou *et al.*	2022	1	(37)
Utsunomyia *et al.*	2022	1	(38)
